# Efficacy of concurrent radiotherapy in patients with locally advanced rectal cancer and synchronous metastasis receiving systemic therapy

**DOI:** 10.3389/fonc.2023.1099168

**Published:** 2023-03-30

**Authors:** Tzu-Chieh Yin, Po-Jung Chen, Yung-Sung Yeh, Ching-Chun Li, Yen-Cheng Chen, Wei-Chih Su, Tsung-Kun Chang, Ching-Wen Huang, Chun-Ming Huang, Hsiang-Lin Tsai, Jaw-Yuan Wang

**Affiliations:** ^1^Division of General and Digestive Surgery, Department of Surgery, Kaohsiung Medical University Hospital, Kaohsiung Medical University, Kaohsiung, Taiwan; ^2^Department of Surgery, Kaohsiung Municipal Tatung Hospital, Kaohsiung Medical University, Kaohsiung, Taiwan; ^3^Division of Colorectal Surgery, Department of Surgery, Kaohsiung Medical University Hospital, Kaohsiung Medical University, Kaohsiung, Taiwan; ^4^Division of Trauma and Surgical Critical Care, Department of Surgery, Kaohsiung Medical University Hospital, Kaohsiung Medical University, Kaohsiung, Taiwan; ^5^Department of Emergency Medicine, Faculty of Post-Baccalaureate Medicine, College of Medicine, Kaohsiung Medical University, Kaohsiung, Taiwan; ^6^Graduate Institute of Injury Prevention and Control, College of Public Health, Taipei Medical University, Taipei, Taiwan; ^7^Department of Surgery, Kaohsiung Municipal Hsiaokang Hospital, Kaohsiung, Taiwan; ^8^Graduate Institute of Clinical Medicine, College of Medicine, Kaohsiung Medical University, Kaohsiung, Taiwan; ^9^Department of Surgery, Faculty of Post-Baccalaureate Medicine, College of Medicine, Kaohsiung Medical University, Kaohsiung, Taiwan; ^10^Department of Surgery, Faculty of Medicine, College of Medicine, Kaohsiung Medical University, Kaohsiung, Taiwan; ^11^Graduate Institute of Medicine, College of Medicine, Kaohsiung Medical University, Kaohsiung, Taiwan; ^12^Department of Radiation Oncology, Kaohsiung Medical University Hospital, Kaohsiung, Taiwan; ^13^Department of Radiation Oncology, Faculty of Medicine, College of Medicine, Kaohsiung Medical University, Kaohsiung, Taiwan; ^14^Department of Radiation Oncology, Kaohsiung Municipal Ta-Tung Hospital, Kaohsiung Medical University, Kaohsiung, Taiwan; ^15^Center for Cancer Research, Kaohsiung Medical University, Kaohsiung, Taiwan; ^16^Pingtung Hospital, Ministry of Health and Welfare, Pingtung, Taiwan

**Keywords:** metastatic rectal cancer, locally advanced rectal cancer, concurrent radiotherapy, primary tumor resection (PTR), systemic chemotherapy, systemic targeted therapy

## Abstract

**Background:**

Neoadjuvant chemoradiotherapy followed by total mesorectal excision is the standard treatment for patients with nonmetastatic locally advanced rectal cancer (LARC). However, for patients with LARC and synchronous metastasis, the optimal treatment strategy and sequence remain inconclusive. In the present study, we evaluated the efficacy and safety of concurrent radiotherapy in patients with *de novo* metastatic rectal cancer who received chemotherapy and targeted therapy.

**Methods:**

We retrospectively reviewed the data of 63 patients with LARC and synchronous metastasis who received intensive therapy at the study hospital between April 2015 and November 2018. The included patients were divided into two groups: RT-CT, those who received systemic chemotherapy with targeted therapy and concurrent radiotherapy (for primary rectal cancer), and CT, those who received only systemic chemotherapy with targeted therapy.

**Results:**

Treatment response was better in the RT-CT group than in the CT group. The rate of primary tumor resection (PTR) was higher in the RT-CT group than in the CT group (71.4% and 42.9%, respectively; *P* = .0286). The RT-CT group exhibited considerably longer local recurrence-free survival (*P* = .0453) and progression-free survival (PFS; from 13.3 to 22.5 months) than did the CT group (*P* = .0091); however, the groups did not differ in terms of overall survival (OS; *P* = .49). Adverse events were almost similar between the groups, except frequent diarrhea, the prevalence of which was higher in the RT-CT group than in the CT group (59.5% and 23.8%, respectively; *P* = .0075).

**Conclusions:**

In the era of biologics, radiotherapy may increase the resectability of primary rectal tumors, reducing the risk of locoregional failure and prolonging PFS. Concurrent pelvic radiotherapy may not substantially improve OS, which is indicated by metastasis. Hence, the resection of the distant metastases may be essential for improving long-term OS. To further determine the efficacy of concurrent radiotherapy, additional prospective, randomized studies must combine preoperative pelvic radiotherapy with PTR and metastectomy to treat patients with stage IV LARC.

## Introduction

Approximately 704 000 new cases of rectal cancer are reported worldwide every year; of them, approximately 20% to 30% present with synchronous metastasis upon initial diagnosis (1). The liver and lungs are the most common sites of metastasis, and approximately 80% of the total cases of stage IV cancer are associated with unresectable metastatic tumor burden (2). Currently, neoadjuvant concurrent chemoradiotherapy (CCRT) followed by total mesorectal excision (TME) is the standard treatment for patients with nonmetastatic locally advanced rectal cancer (LARC). This approach results in pathological downstaging and ensures improved local control, longer disease-free survival (DFS), and tolerable toxicity (3–7). Short-course preoperative radiotherapy also reduces the risk of local failure in patients receiving TME (8, 9).

Owing to the advancement of chemotherapy and biologics, therapeutic outcomes in patients with metastatic colorectal cancer (mCRC) have improved (10–12). Highly aggressive treatment of metastatic diseases, particularly colon cancer with liver metastasis, with hepatic resection and various regional therapy improves mCRC and prolongs overall survival (OS) (13–16).

To the best of our knowledge, the optimal treatment strategy and sequence for patients with LARC with *de novo* metastasis have not been standardized or documented. The potential benefit of concurrent radiotherapy in this population remains unclear and may be overshadowed by the effects of multiagent systemic therapy. Thus, in the present study, we evaluated the efficacy and safety of concurrent radiotherapy in patients with stage IV LARC receiving systemic chemotherapy and targeted therapy.

## Materials and methods

**Figure 1:**
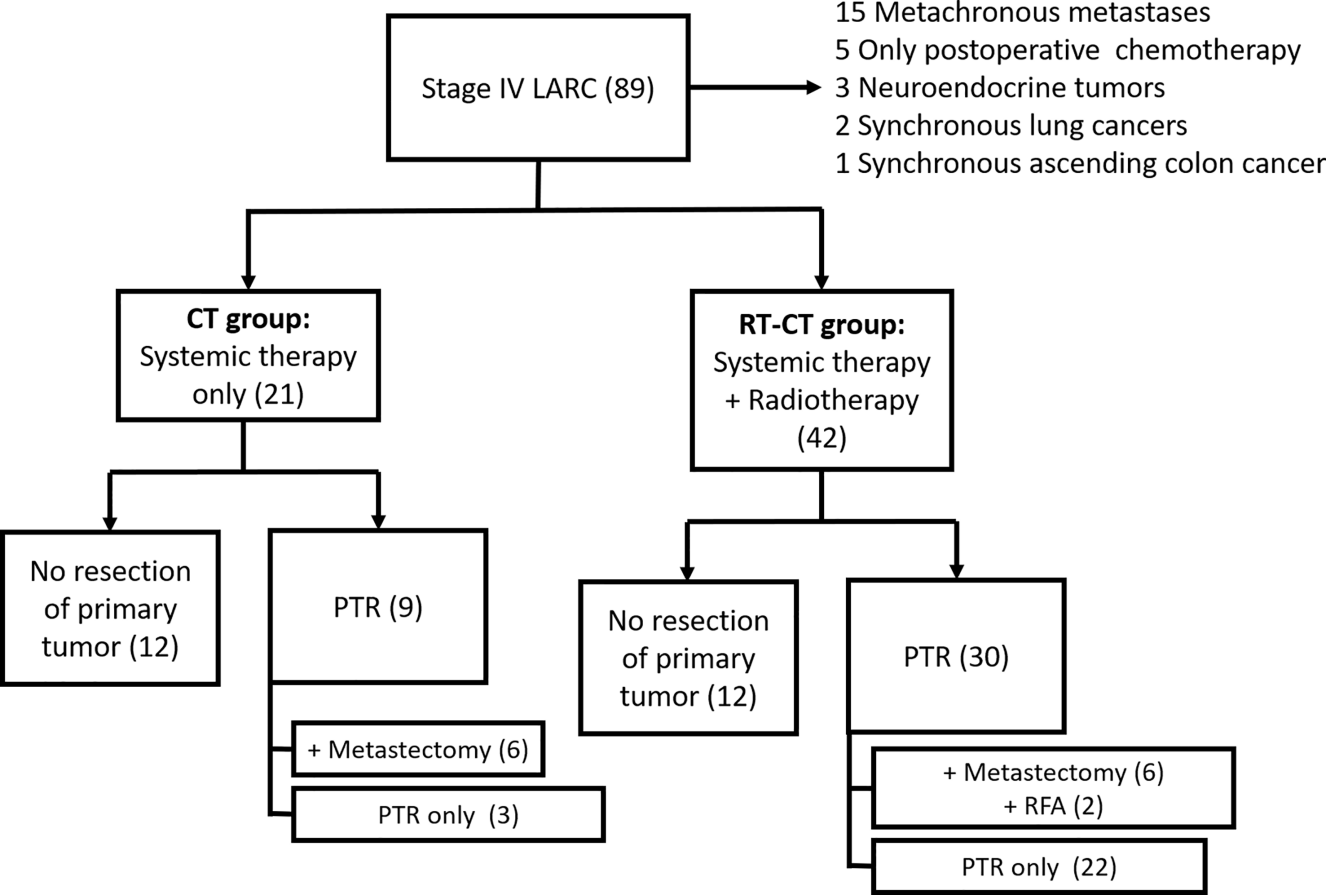
Consort Diagram of Data Collection Process. LARC, locally advanced rectal cancer; PTR, primary tumor resection; and RFA, radiofrequency ablation.

We retrospectively reviewed the data of 63 patients with *de novo* metastatic LARC who underwent intensive therapy at our institution between April 2015 and November 2018. **Figure 1** illustrates the data collection process. This study was approved by the Institutional Review Board of Kaohsiung Medical University Hospital, Taiwan (approval number: KMUHIRB-E(II)-20220041). The inclusion criteria for patient selection were as follows: diagnosis of T3 or T4 and/or N1 or N2 rectal cancer, presence of systemic metastasis, and ongoing systemic chemotherapy. Patients with synchronous secondary cancer, histological malignancy other than adenocarcinoma, or metachronous metastasis or those receiving only postoperative chemotherapy were excluded from this study. The included patients were divided into two groups: RT-CT and CT. The RT-CT group comprised patients who received systemic chemotherapy with targeted therapy and concurrent radiotherapy (for primary rectal cancer), whereas the CT group comprised patients who received only systemic chemotherapy with targeted therapy. Treatments were selected by surgeons or radiation oncologists.

All patients underwent initial workups, which involved taking their medical history, physical examinations, laboratory examinations, carcinoembryonic antigen (CEA) testing, diagnostic colonoscopy, and chest to pelvic computer tomography for preoperative clinical staging. TNM classes were defined in accordance with the criteria outlined by the American Joint Commission on Cancer (AJCC)/International Union Against Cancer (17). Pelvic magnetic resonance imaging (MRI) was performed to evaluate the local status of the primary rectal tumor. To evaluate therapeutic response, MRI was performed again 8 to 10 weeks after pelvic radiotherapy and/or repeatedly performed every 3 months thereafter before primary tumor excision (PTR). Computed tomography was performed at 2- to 3-month intervals to evaluate the progression of distant metastasis and the patients’ response to systemic therapy.

The patients received biweekly systemic therapy comprising chemotherapy with 5-fluorouracil, leucovorin, and irinotecan and targeted therapy with monoclonal antibody against vascular endothelial growth factor (anti-VEGF; bevacizumab) or epidermal growth factor receptor (anti-EGFR; cetuximab or panitumumab). *KRAS* and *NRAS* mutations were detected at diagnosis. The dose of irinotecan was in accordance of *UGT1A1* polymorphism and was reduced by 20% during the addition of concurrent radiotherapy (12, 18). The interval between the last dose of bevacizumab and elective surgery was at least 5 weeks, and bevacizumab was restarted at least 5 weeks postoperatively. Patients who underwent PTR subsequently received chemotherapy and targeted therapy. Long-course radiotherapy was concurrently administered with and at the beginning of systemic therapy in the RT-CT group in accordance with the procedure described in a previously published study (19). The total dose of radiation was 45 to 50.4 Gy (delivered in 25 to 30 fractions). Three-dimensional conformal or intensity- modulated radiation therapy was used for external-beam irradiation.

The response to systemic therapy and radiotherapy was evaluated on the basis of the Response Evaluation Criteria in Solid Tumors (RECIST) (20). Complete response (CR) was defined as the disappearance of all target lesions, whereas partial response (PR) was defined as a ≥30% decrease in the sum of the longest diameters of target lesions from the baseline value. Progressive disease (PD) was defined as a ≥20% increase in the sum of the longest diameters of target lesions from the value recorded at the initiation of treatment or the appearance of ≥1 new lesions. Stable disease (SD) was defined as neither PR nor PD.

The decision to perform surgery for PTR and the timing of surgery depended on the objective outcome of primary tumors and the control of distant metastases after neoadjuvant therapy. In all patients who underwent PTR, TME was performed through conventional laparotomy or minimally invasive surgery (MIS). The procedures were low anterior resection (LAR), intersphincteric resection (ISR), and abdominal perineal resection (APR). Colostomy was performed if the patients were at risk of total lumen obstruction or bowel rupture or when they underwent PTR and were at risk of anastomotic insufficiency (defunctioning stoma). Colostomy was taken down approximately 3 months after PTR (21). The options for liver-directed therapy were the surgical resection of liver metastases and radiofrequency ablation (RFA).

Postoperative and follow-up surveillance involved routine history taking, physical examinations, CEA testing, and CT at 3-month intervals. Annular colonoscopy was performed and positron emission tomography was executed (if needed). Local recurrence (LR) was defined as recurrence in the pelvic cavity or bowel lumen near an anastomosis. LR-free survival (LRFS) was defined as the interval between PTR and the first radiographic evidence of LR. Progression-free survival (PFS) was defined as the interval between the initiation of treatment and PD or the recurrence of distant metastasis. OS was defined as the between-diagnosis and all-cause death or final follow-up.

We collected data regarding the patients’ demographics and tumor characteristics, namely age, sex, TNM stage, body mass index (BMI), tumor location (distance between a tumor’s caudal margin and anal verge), tumor size, synchronous metastatic site, *RAS* mutation status, and presence of comorbidities. Data regarding treatment and response were biologics used, chemotherapy cycles, and RECIST findings for primary tumors and metastases. Perioperative data and surgical outcomes comprised the records of PTR, curative resection of metastases, site of metastectomy, procedures and methods performed for PTR, physical status based on the classification system of the American Society of Anesthesiologists, preservation of the anal sphincter, addition of defunctioning stoma, and nonclosure of stoma. Histopathological characteristics comprised the status of surgical margin; rate of R0 resection; rate of pathological CR (pCR); histological grading of differentiation; pathological stage of disease; number of harvested lymph nodes; lympho-vascular invasion (LVI), and perineural invasion. The tumor regression grade (TRG) was assessed using the guidelines of the AJCC (22).

Adverse events (AEs) associated with systemic therapy, radiotherapy, and surgical complications were evaluated using the US National Cancer Institute Common Terminology Criteria for Adverse Events (version 4.0; http://ctep.cancer.gov/reporting/ctc.html). AEs associated with systemic therapy were hematologic (e.g., anemia, leukopenia, and thrombocytopenia) and nonhematologic (e.g., nausea or vomiting, diarrhea, fatigue, mucositis, peripheral neuropathy, skin manifestations, alopecia, infection, abnormal liver function, and bowel perforation) events. AEs associated with radiotherapy primarily were radiation dermatitis. Surgical complications were defined as complications developed within 30 days after PTR.

Data were analyzed using JMP for Windows (version 16.0; SAS Institute, Cary, NC, USA). Continuous variables are presented in terms of median and interquartile region (IQR) values, and dichotomous variables are presented in terms of number and percentage values. Between-group comparisons were performed using the χ2 test for categorical variables and Student’s *t* test for quantitative variables. A *P* value of ≤.05 was considered statistically significant. Survival plots (LRFS, PFS, and OS) were constructed using the Kaplan–Meier method, and a log-rank test was used to compare the groups in terms of time-to-event distribution.

## Results

A total of 89 patients were initially identified; of them, 15 had metachronous metastasis, 5 received only postoperative chemotherapy, 3 had neuroendocrine tumors, 2 had synchronous lung cancer, and 1 had synchronous ascending colon cancer (**Figure 1**). After the exclusion of these patients, 63 patients remained for our analysis. Of them, 42 received systemic chemotherapy with targeted therapy and concurrent radiotherapy; they constituted the RT-CT group. The remaining 21 patients received only systemic chemotherapy with targeted therapy and constituted the CT group. In the RT-CT group, 30 patients (71.4%) underwent PTR, whereas 12 received no surgery for primary rectal tumor after radiotherapy. A total of 6 patients underwent curative resection of metastases (3 underwent partial hepatectomy for liver metastases, whereas the remaining 3 underwent lobectomy for lung metastases), and 2 patients underwent RFA for liver metastases. In the CT group, 9 (42.9%) underwent PTR, whereas 12 did not. Of the 9 patients, 6 underwent staged metastectomy (2 patients underwent partial hepatectomy, whereas 4 patients underwent lung lobectomy) after PTR. The patients were followed up until their death, final follow-up, or March 2022.

**Table 1** summarizes the patients’ demographics and tumor characteristics. Not surprisingly, tumor location was more low-lying in the RT-CT group than in the CT group (*P* = .0011); 21.4% of the patients in the RT-CT group had a tumor location of <5 cm; this proportion was 4.8% in the CT group. *KRAS* or *NRAS* mutation was detected in 15 (35.7%) patients in the RT-CT group, which was slightly more than the proportion noted on the CT group (3 patients; 14.3%; *P* = .0904). The groups did not differ considerably in terms of age, sex, clinical stage, tumor size, BMI, *BRAF* mutation status, or the presence of comorbidities (all *P* >.05). The most frequent site of synchronous metastasis was the liver in the RT-CT group (27 patients; 64.3%), followed by the lungs. 12 (57.1%) patients in the CT group exhibited liver or lung metastasis.

**Table 1:** Demographics of patients with stage IV locally advanced rectal cancer and the characteristics of their disease in the RT-CT^1^ and CT^2^ groups.

	RT-CT (N = 42)	CT (N = 21)	*P-*value
**Age, median (IQR)**	62 (54 – 68)	58 (54 - 68)	0.69
**Male (%)**	27 (64.3)	12 (57.1)	0.89
**BMI, median (IQR)**	24.1 (22.3 – 27)	22.3 (18.8 – 25.1)	0.13
**Clinical TNM stage IVa/IVb/IVc (%)**	20/20/2 (47.6/47.6/4.8)	8/9/4 (38.1/42.9/19.1)	0.21
cT1/cT2/cT3/cT4 (%)	0/1/26/15 (0/2.4/61.9/35.7)	0/1/13/7 (0/4.8/61.9/33.3)	0.88
cN0/cN1/cN2 (%)	2/13/27 (4.8/31.0/64.3)	1/9/11 (4.8/42.9/52.4)	0.64
cM1a/cM1b/cM1c (%)	20/20/2 (47.6/47.6/4.8)	8/9/4 (38.1/42.9/19.1)	0.21
**Tumor location**			0.0011*
<5 cm	9 (21.4)	1 (4.8)	
≧5 cm, < 10 cm	17 (40.5)	2 (9.5)	
≧10 cm	11 (26.2)	16 (76.2)	
NS	5 (11.9)	2 (9.5)	
**Tumor size, median (IQR)**	4.7 (3.4 – 7.3)	5.4 (4.9 – 6.2)	0.56
**Metastases site**			–
Liver (%)	27 (64.3)	12 (57.1)	
Lung (%)	15 (35.7)	12 (57.1)	
Non-regional lymph nodes (%)	13 (31.0)	5 (23.8)	
Peritoneum (%)	2 (4.8)	4 (19)	
Spine (%)	2 (4.8)	1 (4.8)	
Adrenal gland (%)	2 (4.8)	2 (9.5)	
Abdominal wall (%)	1 (2.4)	0	
Ovary (%)	0	2 (9.5)	
Bone (%)	1 (2.4)	0	
***KRAS* or *NRAS* mutant (%)**	15 (35.7)	3 (14.3)	0.0904
***BRAF* Mutant (%)**	0	1 (4.8)	0.15
**Comorbidity (%)**	27 (75)	11 (68.8)	0.64
**Follow up, median (IQR)**	28.1 (19.8 – 36.6)	24.5 (16.1 – 32.6)	0.27

^1^Group receiving systemic chemotherapy with targeted therapy plus concurrent radiotherapy.

^2^Group receiving systemic chemotherapy with only targeted therapy.

BMI, body mass index; NS, not stated; WD, well differentiated; MD, moderately differentiated; and PD, poorly differentiated.

*P< .05.

In both groups, most patients received bevacizumab (**Table 2**). In the RT-CT group, 26 patients (61.9%) received bevacizumab, and 14 (33.3%) received cetuximab. A total of 13 (61.9%) and 7 (33.3%) patients in the CT group received bevacizumab and cetuximab, respectively. The RT-CT and CT groups received 14 (median; IQR, 9 to 16) and 12 (IQR, 9 to 13) cycles of chemotherapy, respectively. The groups did not differ substantially in terms biologics used or systemic therapy cycles (both *P* >.05). A total of 12 patients (28.6%) in the RT-CT group were at a risk of total lumen obstruction before or during treatment; loop colostomy was performed to avoid such a situation. In the CT group, 11 (52.4%) patients underwent loop colostomy. The RT-CT group exhibited no increased tendency of acute bowel obstruction after the addition of concurrent radiotherapy to their systemic therapy regimen (*P* = .0663). The response rate (CR + PR) of primary rectal tumor was significantly higher in the RT-CT group than in the CT group (73.8% and 47.6%, respectively; *P* = .0398). The disease control rate (CR + PR + SD) of distant metastases was similar between the RT-CT and CT groups (88.1% and 85.7%, respectively; *P* = .63); distant metastasis remained at-least stable during the first-line therapy in 37 patients in the RT-CT group and 18 patients in the CT group (*P* = .63).

**Table 2:** Comparison of between the RT-CT^1^ and CT^2^ groups in terms of treatment and response.

	RT-CT (N = 42)	CT (N = 21)	*P-*value
**Target therapy agent**			1
Anti-EGFR (%)	16 (38.1)	8 (38.1)	
Anti-VEGF (%)	26 (61.9)	13 (61.9)	
**Chemotherapy cycles, median (IQR)**	14 (9 – 16)	12 (9 – 13)	0.11
**Stomy for lumen obstruction (%)**	12 (28.6)	11 (52.4)	0.0663
**Response rate of primary tumor (CR + PR) (%)**	31 (73.8)	10 (47.6)	0.0398*
**Disease control rate of metastases (CR + PR + SD) (%)**	37 (88.1)	18 (85.7)	0.63

^1^Group receiving systemic chemotherapy with targeted therapy plus concurrent radiotherapy.

^2^Group receiving systemic chemotherapy with only targeted therapy.

EGFR, epidermal growth factor receptor; VEGF, vascular endothelial growth factor; CR, complete response; PR, partial response; and SD, stable disease.

*P < .05.

The proportion of patients who underwent PTR was significantly higher in the RT- CT group than in the CT group (*P* = .0286; **Table 3**). A total of 30 (71.4%) patients in the RT-CT group underwent PTR after receiving concurrent radiotherapy with systemic therapy, whereas 9 patients (42.9%) in the CT group underwent PTR after receiving systemic therapy. In the RT-CT group, 24 (80%), 4 (13.3%), and 2 (6.7%) patients underwent LAR, ISR, and APR, respectively. All patients in the CT group received LAR. MIS was performed in 16 (53.4%) and 7 (77.8%) patients in the RT-CT and CT groups, respectively; the groups did not differ in terms of surgical method (*P* = .34). The rates of anal preservation in the RT-CT and CT groups were 93.3% and 100%, respectively. Defunctioning stoma was created during PTR performed in 13 patients (43.3%) in the RT-CT group and 1 patient (11.1%) in the CT group. This was expected because the number of patients with low-lying rectal cancer was higher in the RT-CT group than in the CT group. Metastectomy or liver-directed local therapy (RFA) was performed in 8 patients (19.1%) in the RT-CT group; of them, 3 underwent partial hepatectomy, 2 underwent RFA, and 3 underwent lung lobectomy. Curative resection of metastases was performed in 6 patients (28.6%) in the CT group; of them, 2 underwent partial hepatectomy, and 4 underwent lung lobectomy. In both groups, metastectomy was performed in a staged manner; the number patients who underwent metastectomy didn’t vary significantly between the groups (*P* = .40).

**Table 3:** Perioperative data and surgical outcomes recorded in the RT-CT^1^ and CT^2^ groups.

	RT-CT (N = 42)	CT (N = 21)	*P*-value
**PTR (%)**	30 (71.4)	9 (42.9)	0.0286*
**Curative resection of metastases (%)**	8^a^ (19.1)	6 (28.6)	0.40
**Site of metastectomy**			–
Liver (%)	5 (11.9)	2 (9.5)	
Lung (%)	3 (7.1)	4 (19.1)	
**Procedures performed for PTR LAR/ISR/APR (%)**	24/4/2 (80/13.3/6.7)	9/0/0 (100/0/0)	0.18
**Methods of PTR Open/MIS (%)**	14/16 (46.7/53.4)	2/7 (22.2/77.8)	0.34
**ASA 2/3/NS (%)**	15/14/1 (50/46.7/3.3)	2/6/1 (22.2/66.7/11.1)	0.27
**Sphincter preservation rate (%)**	28 (93.3)	9 (100)	0.30
**Defunctioning stoma with PTR (%)**	13 (43.3)	1 (11.1)	0.13
**Non-closure of stoma (%)**	17 (40.5)	6 (28.6)	0.63

^1^Group receiving systemic chemotherapy with targeted therapy plus concurrent radiotherapy.

^2^Group receiving systemic chemotherapy with only targeted therapy.

PTR, primary tumor resection; RFA, radiofrequency ablation; LAR, low anterior resection; ISR, intersphincteric resection; APR, abdominal perineal resection; MIS, minimally invasive surgery; and ASA, American Society of Anesthesiologists.

*P < .05.

a Including 2 patients who underwent radiofrequency ablation.

**Table 4** summarizes the histopathological characteristics of primary tumors. The status of resection margin in terms of distal resection margin and circumferential resection margin (CRM) was similar between the groups. A total of 2 patients in the RT-CT group and 1 patient in the CT group exhibited positive CRM. The rate of R0 resection in the RT-CT and CT groups was 93.3% and 88.9%, respectively. In the RT-CT group, 4 patients exhibited pCR (13.3%) after concurrent radiotherapy and TME; this number was 1 in the CT group (*P* = .67). TRGs 0, 1, 2, and 3 were detected in, respectively, 4 (13.3%), 7 (23.3%), 14 (46.7%), and 5 (16.7%) patients in the RT-CT group and 1 (12.5), 1 (12.5%), 3 (37.5%), and 3 (37.5) patients in the CT group (*P* = .65). After preoperative radiotherapy, tumor size markedly reduced with a median size of 2.5 cm compared with 3.5 cm without radiotherapy (*P* = .0105). Regarding pathological stages, the groups did not vary significantly in terms of ypT stage (*P* = .64). However, significant between-group differences were noted in terms of ypN stage (*P* = .0197); the proportion of patients with ypN2 stage tumor was higher in the CT group (33.3%) than in the RT-CT group (6.7%). The number of harvested lymph nodes was lower in the RT-CT group (median number, 7) than in the CT group (median number, 16; *P* = .0365).

**Table 4:** Comparison between the RT-CT and CT groups in terms of the histopathologic characteristics of resected primary tumors.

	RT-CT (N = 30)	CT (N = 9)	*P*-value
**DRM, median (IQR)**	2.0 (1.4 – 2.6)	1.8 (1 – 2.2)	0.35
**DRM involvement (%)**	0	0	–
**CRM, median (IQR)**	1.5 (0.4 – 2.5)	0.9 (0.7 – 3.3)	0.98
**CRM involvement (%)**	2 (6.7)	1 (11.1)	0.67
**R0 resection (%)**	28 (93.3)	8 (88.9)	0.67
**pCR (%)**	4 (13.3)	1 (11.1)	0.86
**TRG 0/1/2/3 (%)**	4/7/14/5 (13.3/23.3/46.7/16.7)	1/1/3/3 (12.5/12.5/37.5/37.5)	0.65
**Tumor size, median (IQR)**	2.5 (1.7 – 3)	3.5 (2.6 – 3.7)	0.0105*
**Histology** **WD/MD/PD/NS (%)**	4/22/2/2 (13.3/73.3/6.7/6.7)	0/8/1/0 (0/88.9/11.1/0)	0.32
**pT stage pT0/pT1/pT2/pT3/pT4 (%)**	4/1/7/16/2 (13.3/3.3/23.3/53.3/6.7)	1/0/1/5/2 (11.1/0/11.1/55.6/22.2)	0.64
**pN stage** **pN0/pN1/pN2 (%)**	19/9/2 (63.3/30/6.7)	6/0/3 (66.7/0/33.3)	0.0197*
**Number of harvested LN, median (IQR)**	7 (5 – 13.2)	16 (10 – 25)	0.0365*
**LVI (%)**	5 (16.7)	2 (22.2)	0.73
**Perineural invasion (%)**	4 (13.3)	4 (44.4)	0.13

^1^Group receiving systemic chemotherapy with targeted therapy plus concurrent radiotherapy.

^2^Group receiving systemic chemotherapy with only targeted therapy.

DRM, distal resection margin; CRM, circumferential margin; pCR, pathologic complete response; TRG, tumor regression grade; NS, not stated; LN, lymph nodes; and LVI, lympho-vascular invasion.

*P < .05.

**Table 5** summarizes the AEs associated with concurrent radiotherapy and systemic therapy. Anemia was identified to be the most common hematologic AE in both the RT- CT (90.5%) and CT (95.2%) groups. In the RT-CT group, the most prevalent nonhematologic AEs were diarrhea (25 patients; 59.5%) and fatigue (18 patients; 42.9%). In the CT group, the leading AEs were nausea/vomiting and fatigue, which were observed in 10 (47.6%) patients. In the CT group, diarrhea (any grade) was noted in only 5 (23.8%) patients, which was significantly less than in the RT-CT group (*P* = .0075). Grade III or IV AEs were not frequently detected. Leukopenia and infectious complications were prominent AEs observed in 7 (16.7%) and 5 (11.9%) patients in the RT-CT group, respectively. 3 (14.3%) patients in the CT group developed leukopenia during the treatment course. Radiation dermatitis was observed in 13 (31%) patients in the RT-CT group. Notably, spontaneous rectal perforation developed during or shortly after preoperative radiotherapy in 3 patients (7.1%), and they immediately underwent loop colostomy. Of them, only 1 underwent subsequent PTR. In patients who received concurrent radiotherapy and underwent PTR, infectious complications and postoperative anastomotic leakage were noted in 3 (10%) and 2 (6.7%) patients despite the creation of defunctioning stoma during PTR. Bevacizumab was the monoclonal antibody used in systemic therapy in all the 3 patients of spontaneous rectal perforation and 2 patients of postoperative anastomotic leakage.

**Table 5:** Adverse effects related to systemic therapy, radiotherapy, and surgical complications in the RT-CT and CT groups.

	Grade III-IV			Any grade		
	RT-CT (N = 42)	CT (N = 21)	*P*-value	RT-CT (N = 42)	CT (N = 21)	*P*-value
Hematologic toxicity						
Anemia	3 (7.1)	2 (9.5)	0.75	38 (90.5)	20 (95.2)	0.49
Leukopenia	7 (16.7)	3 (14.3)	0.81	29 (69.1)	12 (57.1)	0.35
Thrombocytopenia	0	0	–	6 (14.3)	2 (9.5)	0.58
Non-hematologic toxicity						
Nausea/vomiting	1 (2.4)	0	0.37	13 (31)	10 (47.6)	0.20
Diarrhea	3 (7.1)	1 (4.8)	0.71	25 (59.5)	5 (23.8)	0.0075*
Fatigue	0	0	–	18 (42.9)	10 (47.6)	0.72
Mucositis	0	0	–	10 (23.8)	6 (28.6)	0.68
Parasthesia	0	0	–	3 (7.1)	1 (4.8)	0.71
Rash acneiform/palmar-plantar erythema	1 (2.4)	0	0.37	8 (19.1)	3 (14.3)	0.63
Alopesia	0	0	–	4 (9.5)	4 (19.1)	0.30
Infection	5 (11.9)	1 (4.8)	0.34	5 (13.9)	5 (11.9)	0.23
Abnormal liver function	2 (4.8)	0	0.20	13 (31)	7 (33.3)	0.85
Bowel perforation	3 (7.1)	0	0.11	3 (7.1)	0	0.11
Radiation dermatitis	0	–	–	13 (31)	–	–
Surgical complications						
Anastomotic leakage	2 (6.7)	0	0.30	2 (6.7)	0	0.30
Infectious complications	2 (6.7)	1 (11.1)	0.67	3 (10)	1 (11.1)	0.92

^1^Group receiving systemic chemotherapy with targeted therapy plus concurrent radiotherapy.

^2^Group receiving systemic chemotherapy with only targeted therapy.

*P < .05.

**Figure 2:**
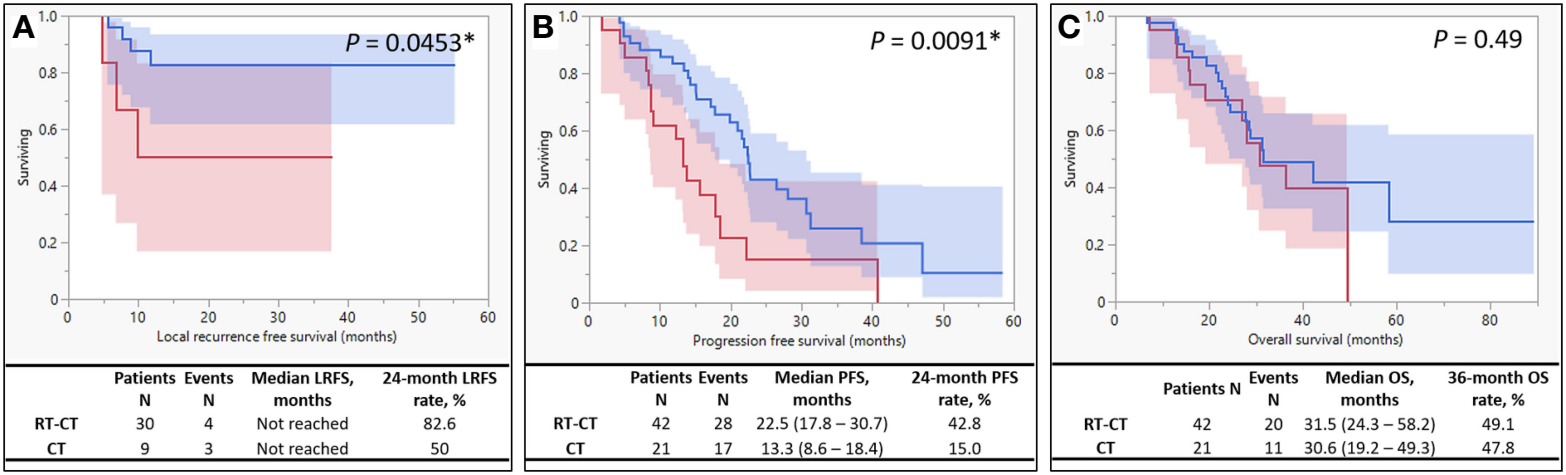
Kaplan–Meier Survival Curves. The survival curve of the RT-CT (systemic chemotherapy with targeted therapy plus pelvic radiotherapy) group is indicated by blue, and that of the CT (systemic chemotherapy with targeted therapy alone) group is indicated by red. **(A)** Local recurrence-free survival, **(B)** progression-free survival, and **(C)** overall survival.

The median follow-up duration was 27 (range, 6.7 to 89.2) months. The 24-month LRFS rates of the RT-CT and CT groups were 82.6% and 50%, respectively (**Figure 2A**). In patients with stage IV LARC who underwent PTR, LRFS was significantly better (*P* = .0453) in those who received concurrent radiotherapy than in those who did not. The median PFS of the RT-CT group was 22.5 months, which was significantly better than that of the CT group (13.3 months; *P* = .0091; **Figure 2B**). However, the 2 groups did not differ significantly in terms of OS (RT-CT group, 31.5 months; CT group, 30.6 months; *P* = .49; **Figure 2C**).

## Discussion

Our findings indicate that patients with relatively low-lying rectal tumors exhibit a high tendency of receiving radiotherapy in addition to systemic therapy even in stage IV of the disease. Although the 2 groups in our study varied in terms of metastatic tumor sites and load, they exhibited similarity in terms of M stage. The addition of concurrent radiotherapy enhanced tumor response. Consistent with the findings of studies on LARC (23) and locally advanced colon cancer (24), in our study, a prolonged interval between preoperative radiotherapy and surgery did not increase the risk of disease progression; this assertion is based on the fact that the disease control rate of distant metastases was noninferior in the RT-CT group. Improved response of primary rectal tumor facilitated PTR after radiotherapy. Histopathologically, no differences were noted between the group in terms of resection margin status, pCR rate, and TRG. However, tumor shrinkage was markedly higher in the RT-CT group than in the CT group. Furthermore, lymph nodes exhibited better response after pelvic irradiation since less ypN2 was obtained in the RT-CT group than in the CT group.

We observed satisfactory local control after concurrent radiotherapy and PTR. The addition of radiotherapy to the systemic chemotherapy regimen increased the rate of 24-month LRFS. It also prolonged (from 13.3 to 22.5 months) the PFS of patients with synchronous metastasis. Few studies have reported similar findings. Concurrent radiotherapy exerted no considerable positive effects on the OS of patients with stage IV LARC. The AEs associated with radiotherapy and systemic therapy were generally tolerable and easily manageable. However, clinicians must consider the risks of spontaneous rectal rupture and anastomotic insufficiency in patients with stage IV LARC receiving simultaneous radiotherapy and targeted therapy, particularly with bevacizumab.

Circulatory tumor cells (CTCs) accelerate micrometastases and are associated with disease progression and survival in breast cancer (25, 26). After preoperative chemoradiotherapy, the proportion of CTCs reportedly decrease in patients with rectal cancer, delaying disease progression (27). Sun et al. revealed considerably lower proportions of CTCs in patients with LARC receiving neoadjuvant CCRT, particularly the responders (28). As expected, we discovered that PFS improved after the addition of concurrent radiotherapy to the current multimodality treatment regimen for LARC with synchronous metastasis. This improvement may also be associated with changes in systemic inflammation and immune function. The neutrophil-to-lymphocyte ratio (NLR) is an indicator of systemic inflammation and may serve as a prognostic factor for various cancers, including rectal cancer (29). A strong correlation has been reported between tumor volume in rectal cancer and NLR (30); the high value of NLR observed in patients with rectal cancer after preoperative radiotherapy has been associated with poor pathological response and survival outcomes (31, 32).

Metastectomy is a key predictor of survival in patients with rectal cancer with metastasis; R0 resection of metastases confers the largest survival benefits (33, 34). In the present study, the improvement in PFS due to additional radiotherapy did not translate to long-term survival. The discrepancy between PFS and OS could be attributed to the low number of patients who underwent curative resection of metastases; in the RT-CT group, only 6 patients underwent metastectomy for liver or lung metastases, and 2 patients underwent RFA. Therefore, the major determinators of OS may depend on the control of distant metastasis. Hence, attempt should still be made for resection of distant metastases to prolong OS.

In patients with mCRC, the precise use of targeted therapy (on the basis of patients’ genetic profiles) and liver-directed therapy results in improved treatment outcomes. In this cohort, late LR become noteworthy, and radiotherapy is a reasonable option for reducing locoregional failure. However, the results in the literature are inconclusive. Kim et al. analyzed data on patients with stage IV rectal cancer with synchronous liver metastasis who underwent TME and liver-directed therapy; LR rate (LRR) was lower in patients receiving postoperative chemoradiotherapy than in those receiving only chemotherapy (35). Fossum et al. demonstrated that neoadjuvant radiotherapy markedly decreased LRR in patients with LARC with resectable liver and/or lung metastasis (36). Chang et al. revealed a trend toward relatively low LRR in patients who underwent PTR treated with postoperative CCRT (37). In their propensity score matching study, Lin et al. indicated improved survival in patients with stage IV rectal cancer when the patients had received CCRT before PTR (34). However, several other studies have reported contradictory findings. A study indicated poor treatment responses and reduced pathological downstaging rates after neoadjuvant radiotherapy in patients with stage IV rectal cancer compared with the findings observed in those with stage II or III disease (38). An et al. reported a nonsuperior LRR in patients who underwent TME and simultaneous metastectomy of limited liver metastases after additional radiotherapy than in those who underwent surgery after only systemic therapy (39). Lee et al. demonstrated that postoperative pelvic radiotherapy improved LRFS only in patients with pT4 disease with metastasis (33). Manyam et al. suggested that preoperative radiotherapy should be avoided in patients with metastatic rectal cancer because the pathological downstaging of rectal cancer for surgical resection is at the expense of increased postoperative complications (40). Consistent with the findings of our study, many studies have reported nonsignificant long-term survival benefits in patients with metastatic rectal cancer who received neoadjuvant or adjuvant radiotherapy, including those who exhibited improved local control (33, 35–41).

In patients with limited liver metastasis burden and satisfactory performance status, prolonged DFS and favorable OS may be achieved after combined liver and colorectal resection (2, 42); PTR with TME should be performed in patients exhibiting good prognosis. However, the optimal management strategy for mCRC with unresectable metastasis remains debatable because of various heterogeneities. The *in-situ* retention of primary tumors in patients with mCRC rarely results in life-threatening events unless complete obstruction, intractable bleeding, or potential tumor perforation is evident. Therefore, the efficacy of PTR in unresectable metastases remains controversial. In patients with asymptomatic mCRC with unresectable metastasis, PTR may be more effective than palliative chemotherapy alone in terms of the superiority of median OS (43). A propensity score matching analysis revealed a 2-year increase in the median OS of patients who underwent PTR (44). In a population-based cohort study including more than 37 000 patients with mCRC who did not undergo metastectomy, PTR in asymptomatic patients was associated with prolonged OS and cancer-specific survival (45).

Except for the low-lying tumor location, patients of better performance status and low metastatic burden appear to be highly likely to receive a multimodality treatment including concurrent radiotherapy and PTR. However, in the present study, the considerable differences in PFS between-group were unlikely solely due to the effects of unadjusted confounders. Unlike in other studies, all the patients included in our study received biologics as part of systemic therapy; this might have controlled metastasis and highlighted the positive effects of concurrent radiotherapy on PFS.

Our study has some limitations, such as the relatively small sample size and between-group heterogeneity in terms of metastatic tumor sites and load. Nevertheless, the finding that concurrent radiotherapy may delay disease progression may help improve the management of patients with LARC with synchronous metastasis.

## Conclusions

The combination of concurrent radiotherapy and systemic therapy may increase primary tumors’ resectability and prolong LRFS in patients with LARC with *de novo* metastasis. Radiotherapy may also substantially improve PFS. However, the resection of distant metastases is recommended to improve OS. In the era of biologics, the combination of preoperative concurrent radiotherapy and subsequent PTR may be a promising multimodality treatment approach for patients with stage IV LARC.

## Data availability statement

The raw data supporting the conclusions of this article will be made available by the authors, without undue reservation.

## Ethics statement

The studies involving human participants were reviewed and approved by Institutional Review Board of Kaohsiung Medical University Hospital, Taiwan (approval number: KMUHIRB-E(II)-20220041). The patients/participants provided their written informed consent to participate in this study.

## Author contributions

T-CY, being the first author of this manuscript, designed this study, analyzed the data, and wrote the manuscript. W-CS, P-JC, T-KC, Y-CC, C-CL, Y-SY, C-WH and C-MH made substantial contributions in terms of the data acquisition, interpretation and statistical analyses, in addition to assisting with the manuscript preparation. H-LT and J-YW, being the corresponding author for this manuscript, also participated in the study design and coordination, in addition to making critical revisions to the manuscript. All authors have reviewed and approved submission of the final version of the manuscript.
